# Age-related increase of thromboxane B_2_ and risk of cardiovascular disease in atrial fibrillation

**DOI:** 10.18632/oncotarget.9826

**Published:** 2016-06-05

**Authors:** Daniele Pastori, Pasquale Pignatelli, Alessio Farcomeni, Cristina Nocella, Simona Bartimoccia, Roberto Carnevale, Francesco Violi

**Affiliations:** ^1^ I Clinica Medica, Atherothrombosis Center, Department of Internal Medicine and Medical Specialties, Sapienza University of Rome, Rome, Italy; ^2^ Department of Public Health and Infectious Diseases, “Sapienza” University of Rome, Rome, Italy; ^3^ Department of Medical-Surgical Sciences and Biotechnologies, Sapienza University of Rome, Latina, Italy

**Keywords:** atrial fibrillation, aging, thromboxane, cardiovascular disease, platelet, Gerotarget

## Abstract

Aging is strictly associated with an increased incidence of cardiovascular events (CVEs) in the general population. Mechanisms underlying the risk of CVEs are still unclear. Platelet activation contributes to the onset of cardiovascular complications. The incidence of atrial fibrillation (AF) increases with age, and the natural history of AF is often complicated by CVEs. We prospectively investigated the relationship between age, urinary thromboxane (Tx) B_2_, which reflects platelet activation, and CVEs in 833 AF patients. Median TxB_2_ level was 120 [66-200] ng/mg of urinary creatinine. At multivariable linear regression analysis, age (B: 0.097, *p*=0.005) and previous MI/CHD (B: 0.069, *p*=0.047) were associated with log-TxB_2_ levels. When we divided our population into age classes (i.e. < 60, 60-69, 70-79, ≥ 80 years), we found a significant difference in TxB_2_ levels across classes (*p*=0.005), with a significant elevation at 74.6 years. During a mean follow-up of 40.9 months, 128 CVEs occurred; the rate of CVEs significantly increased with age classes (Log-rank test, *p* < 0.001). TxB_2_ levels were higher in patients with, compared to those without, CVEs in patients aged 70-79 (*p* < 0.001) and ≥ 80 (*p* = 0.020) years. In conclusion, TxB_2_ levels enhance by increasing age, suggesting that platelet activation contributes to CVEs in elderly patients with AF.

## INTRODUCTION

Cardiovascular events (CVEs) represent the main cause of morbidity and mortality in the elderly population [1]. Several mechanisms have been proposed so far to explain the age-related incidence of CVEs. Thus, the prevalence of cardiovascular risk factors, such as smoking, hypertension and diabetes increases in elderly people. Of note is that despite increasingly effective cardiovascular preventive strategies, a portion of patients still experience cardiovascular complications.

Platelets play a key role in the process of athero-thrombosis as indicated by primary and secondary interventional trials with aspirin, which irreversibly acetylates COX1 so preventing platelet formation of the pro-aggregating eicosanoid Thromboxane (TxB) A_2_. These trials demonstrated, a significant reduction of CVEs in patients treated with aspirin [2, 3]. While experimental studies demonstrated a relationship between platelet activation and aging in animals [4], data in humans are inconclusive because of unreliable methods to assess platelet activation or small sample size [5–7].

Atrial fibrillation (AF) is an interesting model to explore this issue. Thus, the natural history of AF is paradigmatic of the relationship between age and CVEs, as aging is associated with enhanced AF prevalence [8] and an abrupt increase of CVEs complications CVEs [9, 10]. We speculated that platelet activation may increase by aging and be correlated with CVEs in AF. To explore this hypothesis we measured the urinary excretion of TxB_2_ which is a reliable marker of *in vivo* platelet activation and is independently associated with CVEs in AF. Aim of the study was therefore to investigate if in AF patients a relationship between aging, urinary TxB_2_ excretion and CVEs does exist.

## RESULTS

Mean age of patients was 72.8±8.4 years, and 45.6% were women; 86.7% had hypertension, 19.2% diabetes, and 15.5% heart failure (HF). Moreover, 15.2% had a previous stroke/transient ischemic attack (TIA), 19.3% a previous myocardial infarction (MI)/coronary heart disease (CHD), and 8.9% smoked.

### TxB_2_ levels and age

Median TxB_2_ level was 120 [66-200] ng/mg of urinary creatinine. At multivariable linear regression analysis, age (B: 0.097, *p* = 0.005) and previous MI/CHD (B: 0.069, *p* = 0.047) were associated with log-TxB_2_ levels, after adjustment for gender, hypertension, previous stroke/TIA, diabetes, HF, smoking and statin treatment.

**Figure 1:**
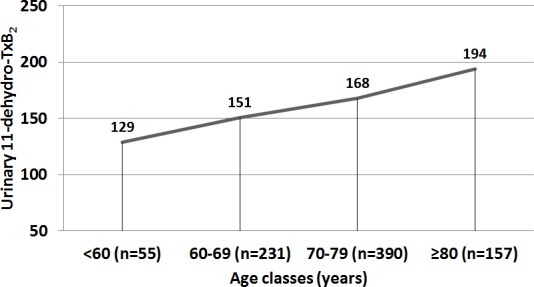
Excretion of urinary 11-dehydro-TxB_2_ according to age classes

Then, we divided our population into age classes (see Methods) and we found a significant difference in TxB_2_ levels across classes (Figure 1, *p* = 0.005), with a significant elevation at 74.6 years (as estimated using shape-restricted B-splines with a change-point).

### TxB_2_ levels, age, and cardiovascular events

**Figure 2:**
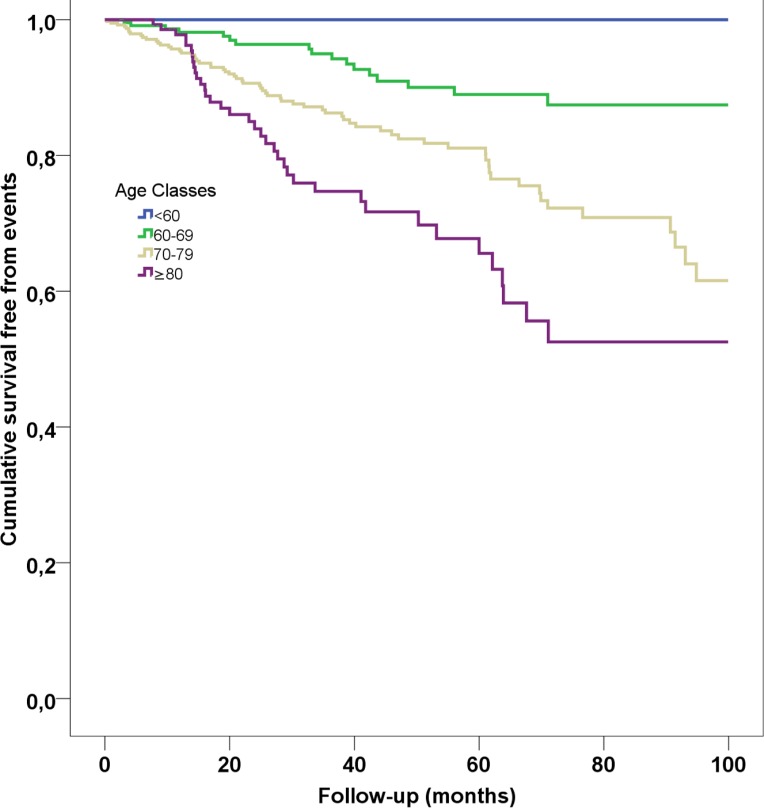
Kaplan-Meier survival curves according to age classes

Comparison of characteristics of AF patients with and without CVEs is reported in Table 1. During a mean follow-up of 40.9±28.8 months, 128 CVEs occurred: 21 fatal/non-fatal ischemic stroke, 25 fatal/non-fatal MI, 19 CHD, 59 vascular deaths, and 4 TIA. Of these, 0 were in class 1, 19 in class 2 (8%), 71 in class 3 (18%) and 38 in class 4 (24%) (Log-rank test, *p* < 0.001, Figure 2).

**Table 1:** Baseline characteristics of the study cohort according to the occurrence of cardiovascular events

Variables	Cardiovascular events		*p* value
	No (*n* = 705)	Yes (*n* = 128)	
**Age (years)**	72.1±8.5	77.0±6.6	<0.001
**Women (%)**	46.1	43.0	0.563
**Smokers (%)**	9.1	7.8	0.737
**CHA**_2_**DS**_2_**-VASc score**^#^	3.0 [2.0-4.0]	4.0 [3.0-6.0]	<0.001
**Arterial Hypertension (%)**	85.7	92.2	0.048
**Diabetes mellitus (%)**	18.2	25.0	0.049
**Heart failure (%)**	13.2	28.1	<0.001
**History of stroke/transient ischemic attack (%)**	12.9	28.1	<0.001
**History of myocardial infarction / coronary heart disease(%)**	16.0	37.5	<0.001
**Statins (%)**	37.4	36.7	0.980
**Thromboxane B**_**2**_ **(ng/mg creatinine)**^#^	110.0 [60.0-187.5]	162.5 [100.0-300.0]	<0.001

# data expressed as median and interquartile range.

TxB_2_ levels were significantly higher in patients with CVEs compared to those without in classes 3 (180 [90-315] *vs*. 100 [58-180], *p* < 0.001) and 4 (162 [120-362] *vs*. 130 [80-200], *p* = 0.020), but not in class 2 (*p* = 0.145).

## DISCUSSION

In this study we analyzed the relationship between platelet activation and aging by classifying AF patients according to decades of age and analyzing the association between platelet activation and CVEs. The novel finding of the present study is that TxB_2_ biosynthesis is closely related with aging, with a progressive increase across the decades and a significant elevation at age > 74 years. Prospective analysis demonstrated that poor vascular outcomes were more frequently detected in AF patients pertaining to classes 3 and 4 (age ≥ 70 years), who disclosed the highest TxB_2_ values.

Previous studies investigated the interplay between platelet activation and aging using *ex vivo* methods to assess platelet function, such as beta-thromboglobulin and platelet factor 4 [5]. Despite a relationship between platelet activation and aging emerged from previous studies, small sample size, lack of follow-up and, overall unreliability, of *ex vivo* tests assessing platelet activation limits definite conclusion.

Differently from these methods, urinary excretion of 11-dehydro- TxB_2_ is a more reliable test to explore platelet activation *in vivo* [11]. However, there are only two studies, which explored the interplay between urinary excretion of 11-dehydro-TxB_2_ with equivocal findings. Thus, Alessandrini et al. [7] studied serum TxB_2_ formation in 177 subjects undergoing laboratory screening for the presence of atherosclerotic risk factors. The Authors found no correlation between TxA_2_ formation and age. However, the overall young age of the cohort (mean age 48 years), and the relative small number of elderly included in the study (only 26 patients aged > 60 years old) may be responsible for this different result. Conversely, Reilly and Fitzgerald [6], in a study involving twenty apparently healthy volunteers, found an increase *in vivo* formation of TxA_2_ in older (> 65 years) than younger (< 40 years) subjects.

Compared to these two previous studies, we included a much larger cohort of patients at risk of cardiovascular disease, which allowed us to assess *in vivo* platelet activation across decades of age. Furthermore, we analyzed the impact of platelet activation in a long-term follow up, and its interplay with aging. Our study has two potentially relevant clinical messages; 1) the increase of platelet activation across decades of age, 2) the close relationship between platelet activation and aging in the occurrence of CVEs. Regarding the first point, we showed that urinary excretion of 11-dehydro-TxB_2_ increases by advancing age, peaking after 70 years. The second finding indicates a close association between aging, platelet activation and CVEs, suggesting that *in vivo* platelet activation may be a mechanism accounting for CVEs in elderly population.

The study has implications and limitations. The present study did not explore the mechanisms linking aging with platelet activation. Experimental study demonstrated that aging is associated with platelet activation and that reactive oxidant species have a role. In particular, Dayal et al showed that platelet production of H_2_O_2_ increases by aging and is associated with platelet activation in mice [4]. The plausibility of this finding relies on the role of H_2_O_2_ to enhance platelet activation *via* activation of COX1 and eventually TxA_2_ [12]. Thus, further study is necessary to investigate the interplay between platelet H_2_O_2_ and CVEs in elderly population.

The clinical implication of this finding is that much more attention should be paid to optimize antiplatelet treatment in the elderly population at risk of CVEs. This issue is even more relevant taking into account that 1) aging negatively influences the ability of low-dose aspirin to inhibit TxB_2_ biosynthesis [13]; 2) aspirin treatment, in addition to anticoagulant therapy, is associated with a significant higher rate of major bleeding in AF [14].

As these data stem from AF population, further study will assess the relationship between TxB_2_, aging and CVEs in other at risk populations. A randomized interventional trial with drugs inhibiting platelet function (i.e. TxB_2_ receptor antagonists) [15], is warranted to explore the potential benefits of modulating platelet function in elderly.

In conclusion, these findings indicate that AF patients over 70 years have significant elevation of platelet activation, which may contribute to the age-related increased risk of CVEs.

## MATERIALS AND METHODS

Baseline urinary 11-dehydro-TxB_2_ (ng/mg of urinary creatinine) levels were measured in 833 non-valvular AF patients treated with vitamin k antagonists (INR range 2.0-3.0). Exclusion criteria: antiplatelet treatment, prosthetic heart valves, cardiac revascularization in the previous year, chronic infections, autoimmune diseases and active cancer.

At baseline, anthropometric data, as well as informations regarding concomitant treatments and comorbidities, were registered. Each patient provided a written informed consent before participating in the study. A urine sample was collected for all patients at baseline (see below).

The occurrence of the first CVE during follow-up was recorded and used for the survival analysis. The composite endpoint of CVEs included: fatal/non-fatal MI and stroke, CHD, TIA and vascular death. The definitions and adjudication of outcomes have been previously reported [16].

### Laboratory analysis

After collection, urine samples were stored at −80°C until use. Urinary excretion of 11-dehydro-TxB_2_ was measured by an ELISA commercial kit (Cayman). Data are expressed as ng/mg creatinine. Intra- and inter-assay coefficients of variation were 4.0% and 3.6%, respectively. Laboratory staff performing TxB_2_ analysis was unaware of clinical characteristics of patients and CVEs.

### Statistical analysis

Categorical variables were reported as counts/percentage and continuous variables as mean (±standard deviation) or median (interquartile range). Student unpaired t test and Mann-Whitney U test were used to compare means and medians, and ANOVA or Kruskall-Wallis test for groups comparison. Stepwise multivariable linear regression analysis was used to assess factors associated with log-TxB_2_ levels, including pre-specified variables. The cumulative incidence of CVEs according to age classes was estimated using Kaplan-Meier survival analysis. *P* values < 0.05 were considered as statistically significant. All tests were two-tailed and analyses were performed using SPSS-18.0, SPSS Inc.
